# Surveillance of Tuberculosis in Taipei: The Influence of Nontuberculous Mycobacteria

**DOI:** 10.1371/journal.pone.0142324

**Published:** 2015-11-06

**Authors:** Chen-Yuan Chiang, Ming-Chih Yu, Shiang-Lin Yang, Muh-Yong Yen, Kuan-Jen Bai

**Affiliations:** 1 International Union Against Tuberculosis and Lung Disease, Paris, France; 2 Division of Pulmonary Medicine, Department of Internal Medicine, Wan Fang Hospital, Taipei Medical University, Taipei, Taiwan; 3 Department of Internal Medicine, School of Medicine, College of Medicine, Taipei Medical University, Taipei, Taiwan; 4 School of Respiratory Therapy, College of Medicine, Taipei Medical University, Taipei, Taiwan; 5 Centers for Disease Control, Department of Health, Taipei, Taiwan; 6 Department of Disease Control and Prevention, Taipei City Hospital, Taipei City Government, Taipei, Taiwan; Hopital Raymond Poincare - Universite Versailles St. Quentin, FRANCE

## Abstract

**Background:**

Notification of tuberculosis (TB) but not nontuberculous mycobacteria (NTM) is mandatory in Taiwan. Partly due to the strict regulation on TB notification, several patients infected with NTM were notified as TB cases. Notification of patients infected with NTM as TB cases can trigger public health actions and impose additional burdens on the public health system. We conducted a study to assess the influence of NTM infection on surveillance of TB in Taipei.

**Methodology/Principal Findings:**

The study population included all individuals with a positive culture for *Mycobacterium* who were citizens of Taipei City and notified as TB cases in the calendar years 2007–2010. Of the 4216 notified culture-positive tuberculosis (TB) cases, 894 (21.2%) were infected with NTM. The average annual reported case rate of infection with NTM was 8.6 (95% confidence interval 7.7–9.4) per 100,000 people. The reported case rate of NTM increased with age in both males and females. The proportion of reported TB cases infected with NTM was significantly higher in females than in males (27.6% vs 17.8%, adjusted OR (adjOR) 1.93, 95% confidence interval (CI) 1.63–2.28); in smear-positive than in smear-negative (23.1% vs 19.2%, adjOR 1.26, 95% CI 1.08–1.47); and in previously treated cases than in new cases (35.7% vs 19.1%, adjOR 2.30, 95% CI 1.88–2.82). The most frequent species was *M*. *avium* complex (32.4%), followed by *M*. *chelonae* complex (17.6%), *M*. *fortuitum* complex (17.0%) and *M*. *kansasii* (9.8%). Of the 890 notified NTM cases assessed, 703 (79.0%) were treated with anti-TB drugs, and 730 (82.0%) were de-notified.

**Conclusions/Significance:**

The influence of NTM on surveillance of TB in Taipei was substantial. Health authorities should take action to ensure that nucleic acid amplification tests are performed in all smear-positive cases in a timely manner to reduce the misdiagnosis of patients infected with NTM as TB cases.

## Introduction

Nontuberculous mycobacteria (NTM) are ubiquitous in the environment.[1, 2] Infection with NTM has been thought to originate from exposure to environmental sources. NTM predominantly affect lung, but they also cause morbidity of soft tissues, lymph nodes, bones, joints, and skin.[3] Because of the new molecular methods for identification and speciation of NTM, the number of NTM species has expanded to more than 150 (http://www.bacterio.net/mycobacterium.html).[4, 5] Studies in several settings have reported that the burden of NTM infection is increasing.[6–12]

Evidence of direct human-to-human transmission of NTM is rare and controversial.[13–16] Infection with NTM has been generally considered a clinical issue that does not require public health intervention. A notification of tuberculosis (TB) is mandatory in many countries, but notification is not required for infection with NTM.[5, 17] However, distinguishing TB from NTM can be difficult. [18–21] Patients infected with NTM may be initially misdiagnosed as having TB, treated with anti-TB drugs and notified as TB cases.

Notification of TB but not NTM is mandatory in Taiwan. [22] A recent study found that completeness of TB notification was as high as 96.5%.[23] Partly due to the strict regulation on TB notification, several patients infected with NTM were notified as TB cases to the Taiwan Centers for Disease Control (CDC). Notification of patients infected with NTM as TB cases can trigger public health actions, including isolation, contact examinations, and arrangement of directly observed therapy, which impose additional burdens on the public health system.

The influence of NTM infection on TB surveillance in Taiwan has not yet been investigated. Therefore, we conducted a study to assess the influence of NTM infection on surveillance of TB in Taipei. The results of this study are reported herein.

## Methods

Objectives: 1) to assess the reported case rate of NTM infection and species identification of NTM among those infected with NTM notified as TB cases in Taipei in 2007–2010 and 2) to assess treatment with anti-TB drugs and de-notification among those infected with NTM who were notified as TB cases in Taipei in 2007–2010.

### Study population

The study population included all individuals with a positive culture for *Mycobacterium* who were citizens of Taipei City and notified as TB cases to the Taiwan CDC in the calendar years 2007–2010.

Lists of all notified TB cases of Taipei City in 2007–2010 were obtained from the National TB Registry at Taiwan CDC. Each TB case had a case management file that was routinely maintained by public health nurses at the Department of Disease Control and Prevention, Taipei City Department of Health, and contained information on the history of TB, sputum examinations (smear, culture, identification, and drug susceptibility testing), body weight, prescription of anti-TB drugs (type of drugs and dosage), outcome of treatment, age, sex, family history of TB, concomitant diseases, smoking, and contact examinations. Information from the National TB Registry and TB case management file was used for this study. No additional data were collected. A structured questionnaire was designed for this study, and a team of 11 individuals was organized for data subtraction, the majority of whom were senior nurses with substantial experience in providing TB services. The questionnaire was discussed, pilot tested, and revised 4 times before it was finalized for data collection.[24]

The overall and age-specific population of Taipei City was obtained from the Ministry of Health and Welfare to calculate the overall and age-specific notification rates of infection with NTM among those who were notified as TB cases to the Taiwan CDC. The reported case rates of NTM were estimated by the number of NTM cases that were notified divided by the population of Taipei City, overall and by age groups. We assume that the reported case rate of NTM followed a Poisson distribution to estimate the 95% confidence interval (CI) of notification rate of infection with NTM. We analyze determinants associated with infection with NTM compared to infection with *M*. *tuberculosis*. We collected data on the species-level identification of NTM. We analyzed factors associated with anti-TB drug treatment and assumed that this treatment was continued before de-notification to estimate the duration of treatment with anti-TB drugs. We assessed the de-notification of those infected with NTM after being notified as TB cases.

### Data entry and analysis

The complete data set was double-entered independently and validated using EpiData Entry 3.1 (EpiData Association, Odense, Denmark). Discrepant records were corrected according to the original data on the questionnaires. STATA Version 12 (StataCorp LP, College Station, TX, USA) was used for statistical analysis. Categorical variables were analyzed using Pearson’s χ2 test. *P* < 0.05 was considered statistically significant. Multivariable logistic regression models were constructed to estimate the odds ratio (OR) and the 95% confidence interval (CI) for the association between the relevant determinants and the outcome of interest, adjusted for covariates.

### Ethics

The study was approved by the proposal review board of Taiwan CDC and funded by the Taiwan CDC. The study utilized routine surveillance data of TB maintained by public health authorities and did not involve any human participation. The questionnaire used for data collection did not contain patient identification information and all patient data collected were anonymized. The patient data were anonymized prior to being accessed by the authors. Therefore, the project was not considered to require review by an ethics committee, and informed consent was waived.

## Results

A total of 6790 TB cases were notified to Taipei health authorities and Taiwan CDC in 2007–2010. Of the 6790 notified TB cases, 4216 (62.1%) were culture positive, and 2574 (37.9%) culture negative. Of the 4216 culture positive cases, 894 (21.2%) were culture positive for NTM.

**Fig 1:**
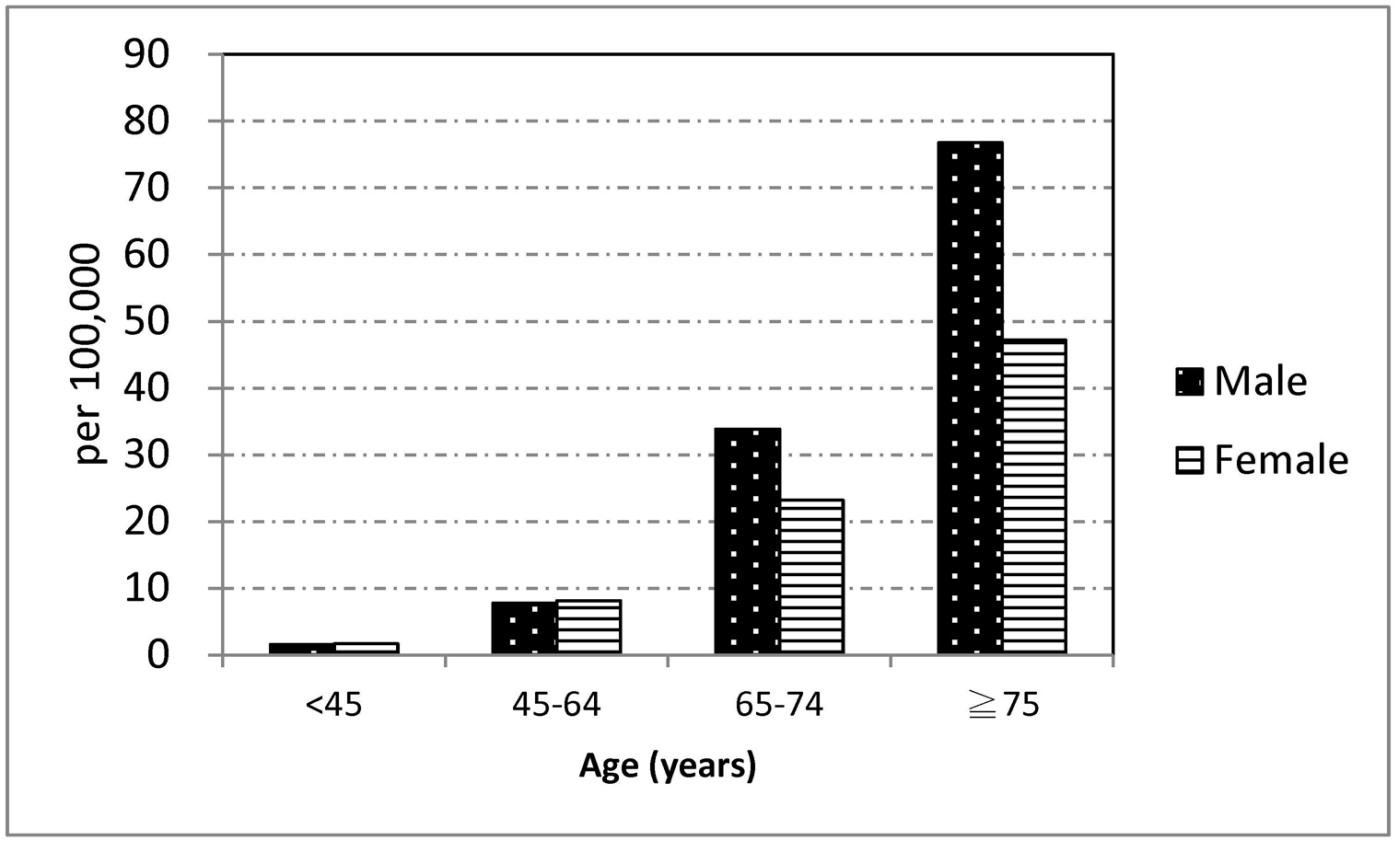
Average notification case rate of nontuberculous mycobacterium in Taipei City, 2007–2010, by sex and age group (per 100,000 inhabitants).

The average annual reported case rate of infection with NTM was 8.6 (95% CI 7.7–9.4) per 100,000 people; it was 9.3 (95% CI 8.2–10.5) per 100,000 people in 2007, 8.3 (95% CI 7.2–9.5) per 100,000 people in 2008, 8.6 (95% CI 7.5–9.8) per 100,000 people in 2009, and 8.0 (95% CI 6.9–9.1) per 100,000 people in 2010. Fig 1 shows average reported case rate of NTM among males and females in Taipei City, 2007–2010, by age group. The reported case rate of NTM increased with age in both males and females. The reported case rate of NTM in those aged 65–74 years was 33.8 (95% CI 22.1–49.6) per 100,000 people among males, which was greater than the 23.2 (95% CI 14.5–35.1) per 100,000 people for females. The reported case rate of NTM for those aged ≧ 75 years was 76.7 (95% CI 58.8–99.2) per 100,000 people among males, which was greater than the 47.2 (95% CI 32.9–66.4) per 100,000 people in females. However, the CI of the reported case rate of NTM among males and females overlapped in both age groups.

Of the 4216 culture positive cases, 4206 had TB case management files that could be located for review, and 10 patients (6 TB cases and 4 NTM cases) did not. Of the 4206 cases, 4078 (97.0%) were pulmonary TB; 3316 (78.8%) were culture positive for *M*. *tuberculosis* and 890 (21.2%) were culture positive for NTM. Of the 3316 patients culture positive for *M*. *tuberculosis*, 67(2.0%) were co-infected with NTM at baseline. Table 1 shows characteristics of notified cases infected with NTM compared with those infected with *M*. *tuberculosis*. The proportion of patients infected with NTM was 22.2% in 2007, 20.5% in 2008, 20.9% in 2009 and 20.9% in 2010 (p = 0.783). The proportion of reported TB cases who were infected with NTM was significantly higher in females than in males (27.6% vs 17.8%, adjusted OR (adjOR) 1.93, 95% CI 1.63–2.28); in those aged 45–64 years (23.8%, adjOR 2.58, 95% CI 1.97–3.40), 65–74 years (24.1%, adjOR 2.63, 95% CI 1.95–3.53), and ≧75 years (22.8%, adjOR 2.47, 95% CI 1.90–3.21) than in those aged <45 years (11.6%); in smear-positive cases than in smear-negative cases (23.1% vs 19.2%, adjOR 1.26, 95% CI 1.08–1.47); in previously treated TB cases than in new cases (35.7% vs 19.1%, adjOR 2.30, 95% CI 1.88–2.82); in those infected with HIV than in those not infected (37.5% vs 21.9%, adjOR 4.67, 95% CI 1.61–13.52); and in non-smokers than in smokers (23.4% vs 14.1%, adjOR 1.46, 95% CI 1.17–1.82).

**Table 1:** Characteristics of reported tuberculosis cases infected with nontuberculous mycobacterium (NTM) compared with those infected with *M*. *tuberculosis*.

	Total	*M*. *tuberculosis*	NTM	Adjusted odds ratio
	N = (col%)	N = (row %)	N = (row %)	(95% CI)
Total	4206(100.0)	3316(78.8)	890(21.2)	
Sex				
Male	2776(66.0)	2281(82.2)	495(17.8)	Ref
Female	1430(34.0)	1035(72.4)	395(27.6)	1.93 (1.63–2.28)
Age(years)				
≤44	774(18.4%)	684(88.4%)	90(11.6%)	Ref
45–64	993(23.6%)	757(76.2%)	236(23.8%)	2.58 (1.97–3.40)
65–74	652(15.5%)	495(75.9%)	157(24.1%)	2.63 (1.95–3.53)
≥75	1787(42.5%)	1380(77.2%)	407(22.8%)	2.47(1.90–3.21)
Smear				
Positive	2096(49.8)	1612(76.9)	484(23.1)	1.26 (1.08–1.47)
Negative*	2110(50.2)	1704(80.8)	406(19.2)	Ref
Type of case				
New	3676(87.4)	2975(80.9)	701(19.1)	Ref
Retreatment	530(12.6)	341(64.3)	189(35.7)	2.30(1.88–2.82)
HIV Infection				
No	3641(86.6)	2845(78.1)	796(21.9)	Ref
Yes	16(0.4)	10(62.5)	6(37.5)	4.67(1.61–13.52)
Unknown	549(13.0)	461(84.0)	88(16.0)	0.74(0.58–0.96)
Smoking				
Never	3147(74.8)	2412(76.6)	735(23.4)	1.46(1.17–1.82)
Ever	906(21.6)	779(85.9)	128(14.1)	Ref
Unknown	152(3.6)	125(82.2)	27(17.8)	1.26(0.79–2.03)

* Nine patients without smear results were classified together as smear negative;

CI: confidence interval.

Of the 890 cases infected with NTM, 500 (56.2%) had species-level identification, but 390 (43.8%) did not. The proportion of NTM cases without species-level identification was 44.7% in 2007, 34.7% in 2008, 37.8% in 2009, and 58.7% in 2010. Table 2 shows the frequency of different species among notified NTM cases in whom species-level identification was available. Of the 500 cases, rapidly growing NTM accounted for 45.2% of all NTM cases. The most frequent species was *M*. *avium* complex (32.4%), followed by *M*. *chelonae* complex (17.6%), *M*. *fortuitum* complex (17.0%), *M*. *kansasii* (9.8%), *M*. *abscessus* complex (9.2%) and *M*. *gordonae* (7.0%).

**Table 2:** Species identification among notified tuberculosis cases infected with nontuberculous mycobacterium, Taipei City, Taiwan, 2007–2010.

		Year			
	Total	2007	2008	2009	2010
	N (col %)	N (col %)	N (col %)	N (col %)	N (col %)
Total	500(100.0%)	135(100.0%)	141(100.0%)	138(100.0%)	86(100.0%)
*M*. *avium* complex	162(32.4%)	44(32.6%)	50(35.5%)	38(27.5%)	30(34.9%)
Rapidly growing	226(45.2%)	56(41.5%)	66(46.8%)	69(50.0%)	35(40.7%)
*M*. *abscessus* complex	46(9.2%)	3(2.2%)	16(11.4%)	20(14.5%)	7(8.1%)
*M*. *chelonae* complex	88(17.6%)	28(20.7%)	19(13.5%)	22(15.9%)	19(22.1%)
*M*. *fortuitum* complex	85(17.0%)	21(15.6%)	30(21.3%)	26(18.8%)	8(9.3%)
Other Rapid growers*	7(1.4%)	4(3.0%)	1(0.7%)	1(0.7%)	1(1.2%)
*M*. *kansasii*	49(9.8%)	12(8.9%)	9(6.4%)	19(13.8%)	9(10.5%)
*M*. *gordonae*	35(7.0%)	14(10.4%)	7(5.0%)	7(5.1%)	7(8.1%)
Others^†^	28(5.6%)	9(6.7%)	9(6.4%)	5(3.6%)	5(5.8%)

*Other rapidly growing: *M*. *flavescens*, *1; M*. *mageritense*, *1; M*. *neoaurum*, 1; species identification not performed, 4.

^†^Others: *M*. *marinum*, 3; *M*. *nonchromogenicum*, 6; *M*. *scrofulaceum*, 2; *M*. *terrae*, *13; M*. *xenopi*, 4.

Of the 890 notified NTM cases, 703 (79.0%) were treated with anti-TB drugs, and 187 (21.0%) were not. Table 3 shows the characteristics of notified NTM cases treated with anti-TB drugs compared with those who were not treated. Those aged <45 years were significantly more likely to be treated with anti-TB drugs compared with those aged ≧75 years. The proportion of patients treated with anti-TB drugs increased in relation to the frequency of positive smear. Those who were smear positive for one specimen (adjOR 1.61, 95% CI 1.09–2.40), two specimens (adjOR 2.19, 95% CI 1.19–4.03) and three specimens (adjOR 2.99, 95% CI 1.51–5.89) were significantly more likely to be treated than were those who were smear negative. Patients infected NTM without species identification (adjOR 2.17, 95% CI 1.45–3.48) and those infected with *M*. *avium* complex (adjOR 2.10, 95% CI 1.27–3.48) were significantly more likely to be treated compared with those infected with rapidly growing mycobacterium. Overall, 26.5% of patients infected with *M*. *kansasii* were not treated.

**Table 3:** Characteristics of notified nontuberculous mycobacterium cases treated with anti-tuberculosis drugs compared with those not treated.

	Total	Not Treated	Treated	Adjusted odds ratio (95% CI)
Total	890(100.0%)	187(21.0%)	703(79.0%)	
Sex				
Male	495(55.6%)	94(19.2%)	400(80.8%)	1.35 (0.96–1.89)
Female	395(44.4%)	92(23.3%)	303(76.7%)	Ref
Age(years)				
≤44	90(10.1%)	14(15.6%)	76(84.4%)	2.12 (1.11–4.06)
45–64	236(26.5%)	43(18.2%)	193(81.8%)	1.44 (0.94–2.21)
65–74	190(21.4%)	48(25.3%)	142(74.7%)	0.92 (0.61–1.41)
≥75	374(42.0%)	82(21.9%)	292(78.1%)	Ref
Frequency of smear positive				
0	392(44.0%)	104(26.5%)	288(73.5%)	Ref
1	267(30.0%)	51(19.1%)	216(80.9%)	1.61 (1.09–2.40)
2	110(12.4%)	18(16.4%)	92(83.6%)	2.19 (1.19–4.03)
3	121(13.6%)	14(11.6%)	107(88.4%)	2.99 (1.51–5.89)
Frequency of culture positive				
1	506(56.8%)	116(22.9%)	390(77.1%)	Ref
2	175(19.7%)	40(22.9%)	135(77.1%)	0.86 (0.55–1.34)
3	209(23.5%)	31(14.8%)	178(85.2%)	1.18 (0.71–1.95)
Type of case				
New	700(78.6%)	144(20.6%)	556(79.4%)	Ref
Previously treated	190(21.4%)	43(22.6%)	147(77.4%)	0.79 (0.53–1.18)
NTM Species				
No speciation	390(43.8%)	66(16.9%)	324(83.1%)	2.17 (1.45–3.24)
*M*. *avium* complex	162(18.2%)	31(19.1%)	131(80.9%)	2.10 (1.27–3.48)
Rapidly growing	223(25.1%)	66(29.5%)	157(70.5%)	Ref
*M*. *kansasii*	49(5.5%)	13(26.5%)	36(73.5%)	1.30 (0.63–2.68)
Others	63(7.1%)	11(17.5%)	52(82.5%)	2.60 (1.25–5.40)

Of the 703 cases treated with anti-TB drugs, 699 (99.4%) were prescribed isoniazid, 694 (98.7%) rifampicin, 681 (96.9%) ethambutol, and 626 (89.0%) pyrazinamide. Further, 139 (19.8%) were treated for ≤15 days, 131 (18.6%) for 16–30 days, 161 (22.9%) for 31–60 days, 83 (11.8%) for 61–90 days and 189 (26.9%) for ≥91 days.

Of the 890 notified NTM cases, 730 (82.0%) were de-notified at a later point in time after notification, but 160 (18.0%) were not. The proportion of patients infected with NTM who were de-notified was 78% in 2007, 80% in 2008, 88% in 2009 and 83% in 2010 (p = 0.019).

## Discussion

NTM are widely distributed worldwide and possess geographical diversity. The global partners in the NTM-Network European Trials Group (NET) framework collected data from 62 laboratories in 30 countries across six continents reported that of the 91 different NTM species isolated from respiratory samples, *M*. *avium* complex predominated in most countries, followed by *M*. *gordonae* and *M*. *xenopi*.[25] Studies have shown that rapid growing mycobacteria are more prevalent than *M*. *gordonae* and *M*. *xenopi* in Taiwan. Lai et al. reported that *M*. *avium* complex (30.0%) were the most frequently isolated organisms, followed by *M*. *abscessus* complex (17.5%), *M*. *fortuitum* complex (13.0%), *M*. *chelonae* complex (9.6%), *M*. *kansasii* (5.6%), and *M*. *gordonae* (5.5%).[7] Our study concurs with that by Lai et al, who showed that *M*. *avium* complex is the most prevalent NTM infection in Taipei, followed by rapidly growing mycobacteria and *M*. *kansasii*.

Lai et al reported that during 2000–2008, the proportion of NTM increased significantly from 32.3% to 49.8%, and the associated disease incidence increased from 2.7 to 10.2 cases per 100,000 patients.[7] Our study revealed that the reported case rate of infection with NTM in individuals who were initially misdiagnosed as TB was approximately 9 per 100,000 people. This finding may not represent the true incidence of infection with NTM in Taipei. Nevertheless, it indicates that the burden of NTM is substantial. The burden of TB in Taiwan has been decreasing. The TB notification rate was 74 per 100,000 people in 2004, which decreased to 53 per 100,000 people in 2012. A recent systemic review reported an increase in the proportion of mycobacterial disease caused by NTM in several settings that was coincident with decreases in TB rates.[26] It is likely that the burden of NTM infection will continue to rise as the epidemic of TB declines in Taiwan.

Our study found that NTM accounted for more than 20% of the TB cases notified to the Taiwan CDC in the study period, and the proportion of patients notified as TB cases who were infected with NTM was even higher among females, the elderly, previously treated TB cases, HIV-infected individuals and non-smokers. The reporting of patients infected with NTM as TB cases to public health authorities has considerable public health implications and imposes substantial direct and indirect costs on patients. Patients infected with NTM are unnecessarily placed in isolation if they are hospitalized and are advised to undertake infection control measure to reduce the non-existent risk of TB transmission at home, with friends or at their work place, which may generate psycho-social stress on patients.

The increased isolation of NTM has imposed challenges on the clinical management of TB.^20^ The majority of the individuals who were infected with NTM were misdiagnosed as TB cases and had been treated with first-line anti-TB drugs. The isolation of NTM from respiratory specimens may reflect a spectrum of clinical conditions from colonization to disease and may not always need treatment. Among those who are clinically judged to require treatment, optimal regimens vary considerably depending on the specific infectious species and usually involve the use of antibiotics that are not part of first line anti-TB drugs.[3, 17, 27, 28] It will be helpful to introduce nucleic acid amplification (NAA) tests as an additional assay for patients with AFB smear-positive sputum.[29] Our study reveals that the proportion of individuals infected with NTM who were treated with anti-TB drugs increased relative to the frequency of positive smear and that those with three positive specimens for AFB were three times more likely to be treated with anti-TB drugs than smear-negative cases. NAA tests performed in a timely manner will be helpful in differentiation between *M*. *tuberculosis* and NTM in smear-positive specimens. If the AFB smear result is positive and the NAA result is negative, the initiation of anti-TB treatment is not indicated before further confirmation.

Our study raised concerns on clinicians’ understanding and training on NTM infection. Pulmonary infection with *M*. *kansasii* is usually associated with significant disease and is commonly treatable with a rifampicin-based regimen (in combination with INH and EMB).[27] Unfortunately, a considerable proportion of patients infected with *M*. *kansasii* in our study were not treated with a rifampicin-based regimen. Our study also highlights the negative influence of NTM on TB surveillance. Although the majority of cases infected with NTM were de-notified at a later point in time after notification, de-notification was delayed, and a considerable proportion was not de-notified.

Our study has several strengths. It is a population-based study that involved all notified TB cases that were culture positive for mycobacterium in Taipei in 2007–2010. The number of cases was considerably large, revealing that a substantial proportion of notified TB cases were in fact infected with NTM. It analyzes the NTM notification rate, assesses factors associated with NTM infection, and reports the management and de-notification of cases infected with NTM, which may provide guidance for policy development. This study also has limitations. The study population consisted of notified TB cases and patients infected with NTM who may not always have been notified. Thus, the notification rate of NTM may not represent the incidence of NTM infection in Taipei. Furthermore, a substantial proportion of cases infected with NTM did not have species identification, likely revealing the weakness of the health care system in providing services to patients infected with NTM. Finally, we did not have a long-term follow-up of outcomes for patients infected with NTM.

## Conclusions

Despite these limitations, we conclude that the influence of NTM on surveillance of TB in Taipei was substantial and should not be neglected. Health authorities should take action to ensure that NAA tests are performed in all smear-positive cases in a timely manner to reduce the misdiagnosis of patients infected with NTM as TB cases. The identification of culture-positive specimens should be performed to assess the requirement of treatment for NTM infection. Finally, health care workers who are involved in reporting and surveillance of TB should be trained; it is essential to ensure that the de-notification of patients infected with NTM occurs as early as possible to reduce the burden on the public health system and ensure the quality of the TB surveillance system.
